# Functional heterogeneity in senescence

**DOI:** 10.1042/BST20190109

**Published:** 2020-05-05

**Authors:** Kristina Kirschner, Nattaphong Rattanavirotkul, Megan F. Quince, Tamir Chandra

**Affiliations:** 1 Institute of Cancer Sciences, University of Glasgow, Glasgow G61 1BD, U.K.; 2 Chakri Naruebodindra Medical Institute, Mahidol University, Samut Prakan 10540, Thailand; 3 MRC Human Genetics Unit, University of Edinburgh, Edinburgh EH4 2XU, U.K.

**Keywords:** heterogeneity, Notch, secondary senescence, senescence, senotherapy

## Abstract

Senescence is a tumour suppressor mechanism which is cell-intrinsically activated in the context of cellular stress. Senescence can further be propagated to neighbouring cells, a process called secondary senescence induction. Secondary senescence was initially shown as a paracrine response to the secretion of cytokines from primary senescent cells. More recently, juxtacrine Notch signalling has been implicated in mediating secondary senescence induction. Primary and secondary senescent induction results in distinct transcriptional outcomes. In addition, cell type and the stimulus in which senescence is induced can lead to variations in the phenotype of the senescence response. It is unclear whether heterogeneous senescent end-points are associated with distinct cellular function *in situ*, presenting functional heterogeneity. Thus, understanding senescence heterogeneity could prove to be important when devising ways of targeting senescent cells by senolytics, senostatics or senogenics. In this review, we discuss a role for functional heterogeneity in senescence in tissue- and cell-type specific manners, highlighting potential differences in senescence outcomes of primary and secondary senescence.

## Introduction

In 1961, Hayflick and Moorhead [1] discovered, albeit in a rather serendipitous manner, that their cell cultures faced proliferative exhaustion after an extended period of time. They suspected that the finite proliferative capacity they observed in cell culture might limit the regenerative capacity of an ageing organism and they, therefore, called the phenomenon cellular senescence. Years later, it was shown that cellular senescence could also be induced in a more acute way, as shown by ectopic activation of oncogenes such as oncogenic H-Ras (proto-oncogene mutated RASG12V) [2]. Ras-induced senescence requires the activation of the Rb transcriptional co-repressor 1 (RB1) and tumour protein 53 (TP53) pathways and has emerged as a stable exit from the cell cycle and an important cell-intrinsic tumour suppressor mechanism [3]. A growing body of evidence has since strengthened Hayflick's suggestion of a pathophysiological role in the loss of function and regenerative abilities in tissues and organs as senescent cells build up over time [4,5]. The clearance of senescent cells leads to improved health outcomes and an extension of healthy lifespan [6]. The accumulation of senescent cells has thus served as a valuable concept to partially explain part of the complex process of ageing and the manifestation of its associated pathologies [7]. In addition to being a barrier to tumourigenesis, senescence has been associated with other physiological processes such as embryonic development [8,9] and wound healing [10,11].

Besides proliferation-related telomere erosion and oncogenic insults, senescence is triggered by various intrinsic and extrinsic factors, such as DNA damage [12], accumulation of reactive oxygen species (ROS) [13], mitogen exposure [14], hypoxia [15], impaired ribosomal biogenesis [16] and mitochondrial dysfunction [17]. Post insult, cells exit the cell cycle by activation of TP53-mediated cyclin-dependent kinase inhibitor 1A (CDKN1A or p21) and/or RB1-mediated negative cell-cycle regulation via p15(INK4B) and p16(INK4A), with the involvement of DNA damage response (DDR) pathways [12]. Fully senescent cells show distinct morphological features; cells often become flat and large, stain positive for senescence-associated β galactosidase (SA-β Gal), whilst nuclear changes occur with the loss of Lamin B1 and chromatin remodelling towards heterochromatic foci, named senescence-associated heterochromatin foci (SAHF) [18–20]. Intrinsic nucleic acid sensing has been implicated in the senescence response, where DNA shed from nuclei, forming cytoplasmic chromatin fragments (CCFs), is degraded in the cytosol [21]. These CCFs are then sensed by the cytosolic DNA-sensing cGAS-STING pathway, resulting in the activation of the senescence-associated secretory phenotype (SASP) [22–25]. Cytosolic nucleic acids might not be the only way to involve innate immune pathways in the senescence response. A recent study shows the involvement of Toll-like receptor 2 (TLR2) via the TP53, p16 and p15 pathways, thereby inducing cell cycle arrest and induction of acute-phase serum amyloids, which are part of damage-associated molecular patterns (DAMP). DAMPs in turn signal through pattern recognition receptors of the innate immune system such as TLR2, thereby controlling the SASP and reinforcing cell cycle arrest during senescence [26].

SASP is one of the most dynamic and complex senescence features. Senescent cells are interacting with themselves and their surrounding microenvironment via the SASP. SASP is characterised as a complex secretome of inflammatory cytokines, chemokines, collagens, metalloproteinases (MMPs) such as interleukin 1 alpha (IL-α) and interleukin 6 (IL-6), together with tumour necrosis factor-alpha (TNF-α), vascular endothelial growth factor (VEGF), insulin-like growth factor (IGF-1), chemokine CXC motif ligands 1 and 2 (CXCL1, CXCL2) and interleukin 8 (IL-8), chemokine CC motif ligand 2 (CCL2) and transforming growth factor-beta (TGF-β) [10,23,24]. Autocrine amplification of senescence occurs via binding of IL-6 to the IL-6 receptor and through chemokines belonging to the CXC family such as IL-8, which bind to the CXCR2 receptor [27].

SASP is capable of inducing senescence in neighbouring cells exposed to the secretome, this is known as paracrine secondary senescence, in contrast to primary senescence established from direct insults to the cell [28,29]. It is thought that this is a mechanism with the purpose of increasing immune cell infiltration and clearance of senescent cells to prevent the development of a pre-neoplastic niche. Failure to clear senescent cells causes systemic inflammation and creates a pro-neoplastic and pro-ageing milieu [30]. Hence, the effect of SASP is thought to be a double-edged sword.

**Figure 1.**
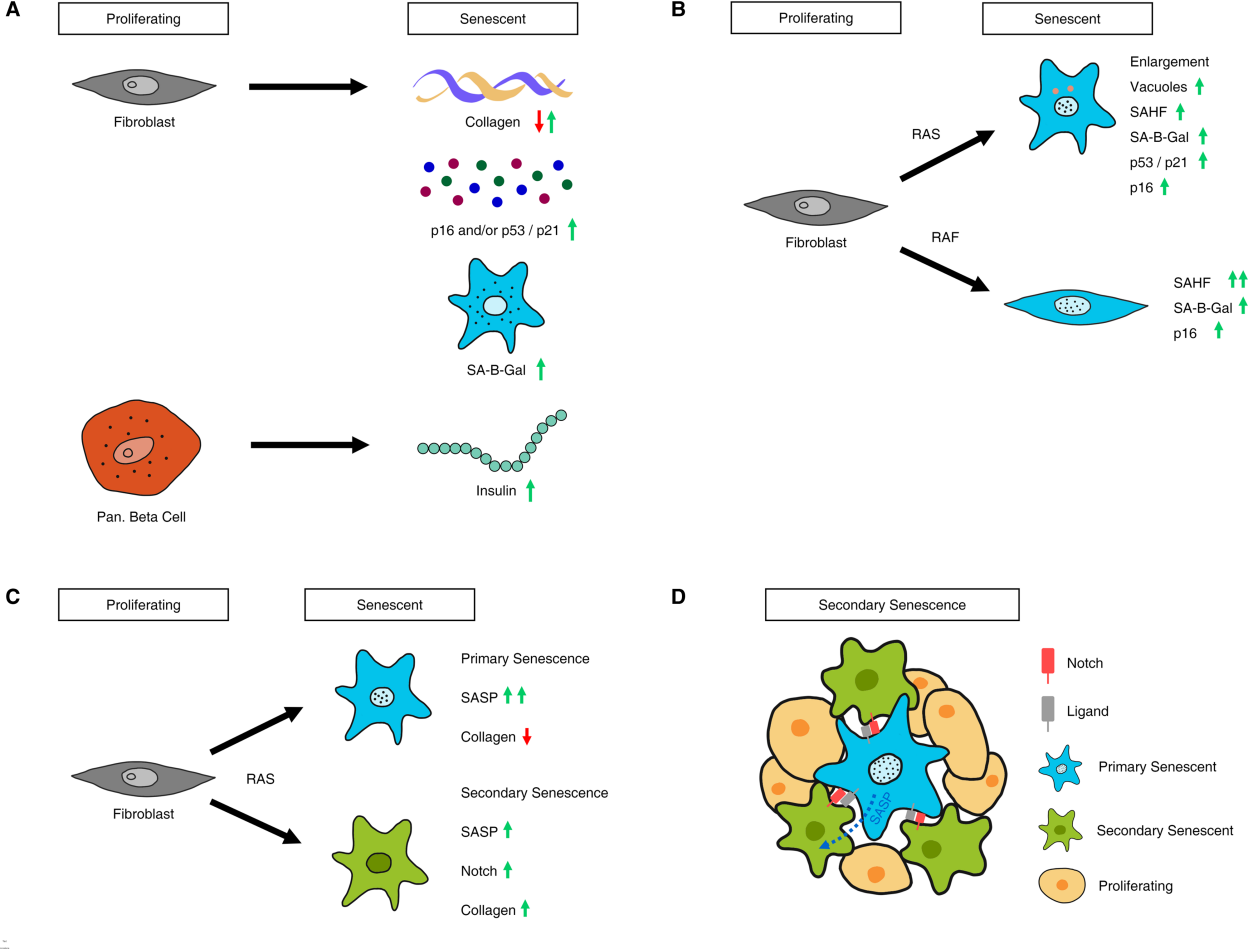
Examples of cellular senescence heterogeneity. (**A**) Senescence is a cell-type specific response. Fibroblasts and pancreatic beta-cells respond by up/down-regulating cell-type specific transcripts and proteins. (**B**) Senescence response varies by inducing oncogene. Although part of the same pathway, RAS and RAF-induced senescence show marked differences in fibroblasts. RAS-induced cells grow in size and form vacuoles. RAF-induced cells show a very high percentage of SAHF positive cells. (**C**) Cell-to-cell variability in oncogene-induced senescence. We have recently shown that RAS induction can lead to a variety of transcriptional end-points. We find an additional, secondary senescent end-point characterised by Notch signaling [30]. (**D**) A composite phenotype results in secondary senescence. Secondary senescence induction can be mediated by primary senescent cells through SASP and Notch.

In recent years, it has become clear that the senescence phenotype is variable depending on the affected cell type, physiological location, the initial causal insult and the molecular pathways being favoured within the cells [31] (Figure 1A). Similarly, SASP composition differs greatly depending on the original insult and cell type, but not all senescent cells produce SASP [32]. This heterogeneity could resemble a scenario with functional diversification between primary senescence in a tissue-specific manner and between primary and secondary senescence within the same organ. The concept of primary and secondary senescence being transcriptionally distinct end-points has provided the biological imperative to investigate the mechanisms that govern such heterogeneity [33]. The direction of future research on the development of senolytic therapies should, therefore, be guided by the pursuit and understanding of this heterogeneity.

## Senescence heterogeneity dependent on cell type and stimulus

Heterogeneity in senescence outcomes in terms of, for example, SASP composition, stability and speed of induction of the phenotype and SAHF formation have been widely described and can be attributed, in part, to the cell type chosen and the senescence inducing agent. Within the scope of this review, we can only illustrate a few examples of this heterogeneity.

### Primary senescence heterogeneity

Human diploid fibroblasts (HDFs) such as IMR90, WI-38 (both embryonic lung fibroblasts) or BJ cells (from foreskin) are standard models in the study of senescence [2,34]. Yet, in IMR90 and WI-38 cells, SAHF formation is more pronounced when given the same stimulus compared with BJ cells [18]. This might be dependent on the levels of p16 pathway activation as senescence in BJ cells typically results in lower levels of p16, with knockdown of RB/p16 abolishing SAHF formation in HDFs [18,35]. It might also depend on the oncogene of choice (Figure 1B). For example, RASG12V-mediated senescence in HDFs leads to robust SAHF formation and an increase in cell and nuclear size [18]. cRAF activation in the same HDFs leads to an even higher SAHF formation, but no increase in cell size [36]. In contrast, weaker cell cycle arrest in the absence of SAHF and SASP was observed upon activation of the phosphatidylinositol-4,5-bisphosphate 3-kinase catalytic subunit alpha/KT serine/threonine kinase (PI3K3CA/AKT) axis downstream of RAS in HDFs [37], pointing to differences in cellular outcomes where parts of the same pathway might be activated to a different degree.

In terms of SASP compositions, ectopic overexpression of Notch Receptor 1 (NOTCH1) in IMR90s led to a highly specific primary senescence-dependent secretome, composed of collagens and TGF-β and a suppression of the RASV12G-driven secretome, normally consisting of IL6/8, CCL2/3 and MMP1/3 [38] (Figure 1C). Moreover, impaired ribosomal biogenesis induced senescence leads to a similarly restricted but yet distinct SASP, characterised by TGF-β [16].

### Secondary senescence heterogeneity

In HDFs, secondary senescence induction was originally thought to be solely explained by paracrine signalling [29]. However, when we analysed single-cell RNA-sequencing data for primary RASV12G-mediated and secondary senescence in IMR90 fibroblasts, we identified a composite SASP signature, partially driven by paracrine factors and partially driven by NOTCH [33]. This composite secondary senescence transcriptome led to a subdued cytokine response via altered SASP elements previously reported for Notch-mediated primary senescence [38] and a marked increase in fibrillar collagen production (Figure 1A,C,D). These alterations could indicate fundamental functional differences between primary and secondary senescence in fibroblasts, leading to context-dependent diverse senescence outcomes and functions. In this context, primary senescence might confer a tumour suppressor activity, where a loss of normal function, namely collagen production, occurs. Secondary senescent cells might, in turn, restore normal fibroblast function in an organ by secreting collagens *de novo* in addition to senescence activation.

More recently, many studies reported the importance of membrane-bound extracellular vesicles such as exosomes and small extracellular vesicles for senescent cell communication and the enforcement of the senescence phenotype. These vesicles facilitate cellular protein transfer to mediate immune surveillance and communication with surrounding cells [39,40]. They can induce paracrine, secondary senescence and are part of the SASP. However, there is also evidence that senescence-associated extracellular vesicles can be pro-tumourigenic, conferring pro-proliferative properties onto some types of cancer cells [41], again illustrating the context dependency of senescence features.

Overall, the huge variety of different secretomes produced by different senescence states and stimuli is likely to create thus far unexplored heterogeneity in a cell type and tissue-specific manner, probably leading to distinct functions and clearance mechanisms. The benefits (clearance, tumour suppression, functional restoration) and detrimental effects (pro-tumourigenic, pro-ageing) of the different SASP components in this context are yet to be determined.

## Senescence heterogeneity in tissue and disease context

### Senescence in the skin

Human naevi are a widely used *in vivo* senescence model. Senescence heterogeneity in melanocytes is partially oncogene dependent. For example, ectopic activation of RASG12V or b-Raf proto-oncogene-mutated V600E (BRAFV600E) in human melanocytes results in oncogene-induced senescence with many shared phenotypes; however, only RASG12V activation leads to an increase in cell size and vacuolization, whereas BRAFV600E activation leads to a significant increase in SAHF positive cells [42]. Ageing of the skin introduces heterogeneity in naevi with different phenotypic changes in primary versus secondary skin senescence. p16 positive, senescent melanocytes accumulate dysfunctional telomeres during ageing [43]. However, neighbouring epidermal cells do not up-regulate p16, but instead display telomere dysfunction and DNA damage, indicating secondary senescence mediation in human naevi from melanocytes to epidermal cells [43]. Secondary senescence induction in the skin might contribute to decreased epidermal regeneration and creates a heterogeneous environment in the ageing skin.

### Senescence in the kidney

Studies to elucidate the function and consequences of a senescent population present in the kidney have also illustrated context dependency in the effect of senescent cells [44], with tubular epithelial cells being most often associated with renal senescence [45]. In kidneys, senescence contributes to fibrosis and tubular atrophy when chronically present in aged tissues [46–48]. Moreover, in glomerular disease, the accumulation of p16-positive cells probably leads to impaired repair and subsequent renal dysfunction [45]. In contrast, in autosomal dominant polycystic kidney disease (ADPKD), where CDKN1A levels are typically low, there is some evidence suggesting that roscovitine-mediated restoration of senescence, as measured by increased SA-β Gal and CDKN1A expression, is beneficial and halts cystic progression [49]. These studies further highlight the complexity of senescence in a tissue context, where senescence outcomes might be specific to disease type.

### Senescence in the pancreas

During ageing, the senescence effector p16 is expressed in pancreatic β islet cells, limiting cell proliferation and possibly regenerative capacity [50]. Senescent pancreatic β islet cells feature other typical senescence characteristics, such as enlarged cell size and SA-β Gal production [51]. Interestingly, p16 expression in senescent pancreatic β islet cells increased glucose uptake and glucose stimulated insulin secretion when exposed to high glucose concentrations, conferring a beneficial effect during ageing [51] (Figure 1A). In young mice and human tissue context, a marked heterogeneity of β islet cells in terms of insulin secretion exists, which persists during ageing, albeit to a lesser degree [52]. However, the levels of p16 and the expression of ageing-associated cell surface proteins, such as insulin growth factor 1 receptor (IGF1R), vary greatly in aged β islet cells within an individual, further suggesting functional heterogeneity in the pancreas during ageing [52], which again, might be dependent on the degree of pathway regulation within a cell.

In contrast to the above described increased insulin secretion during ageing, another study showed that type 2 diabetes insulin resistance leads to increased proliferation of β islet cells and subsequent senescence. Here, as a consequence, the insulin release from senescent β islet cells decreases, thereby contributing to the pathogenesis of type 2 diabetes [53]. In addition, in type 1 diabetes, elimination of a subset of senescent β islet cells was sufficient for disease prevention [54]. Clearly, in diabetes, islet cell senescence contributes to disease pathogenesis and might be a therapeutic target, whereas in an ageing context, islet cell senescence might be partially beneficial.

### Wound healing and fibrosis in tissue context

Senescence-mediated wound healing and fibrosis has been reported in several tissues, with beneficial and detrimental aspects of senescence.

Beneficial aspects of senescence-mediated fibrosis were initially reported in the context of acute liver damage where activated stellate cells underwent senescence [55]. These senescent hepatic stellate cells displayed down-regulation of genes encoding extracellular matrix components with simultaneous up-regulation of extracellular matrix degrading enzymes. Moreover, genes known to stimulate immune surveillance for senescent cell clearance were also up-regulated [55]. This led to controlled, temporary fibrosis during acute liver damage, followed by clearance of senescent cells. However, chronic liver damage was associated with proliferating, activated stellate cells and extensive deposition of extracellular matrix proteins, eventually leading to cirrhosis and liver failure [55]. In addition, studies in idiopathic pulmonary ﬁbrosis highlighted the detrimental effects of pulmonary senescence, partially by secretion of pro-fibrotic lipids, such as leukotrienes [56,57]. The context, but also the kinetics in which cell senescence occurs are, therefore, clearly important factors to determine beneficial and detrimental effects and should be considered more widely when targeting senescent cells.

The importance of kinetics is nicely demonstrated during wound healing, where skin senescence plays a role in fibroblast-mediated collagen deposition and wound closure in young mice. This beneficial effect of senescence is part of an integral interplay between senescent endothelial cells and fibroblasts, with transient up-regulation of p16, CDKN1A and SA-β Gal in both cell types [11]. Senescent fibroblasts produce VEGF, platelet-derived growth factor-AA (PDGF-AA) and SASP, thereby promoting tissue repair. VEGF, in turn, stimulates endothelial proliferation and revascularisation, allowing the transport of fibrinogens and coagulation factors to the wound [11]. The elimination of senescent cells from wounds in mouse models decreased the time to wound closure. A study by Jun and Lau [58] elucidated the mechanism in which extensive fibrosis during wound closure is curbed. They identified the matricellular protein cellular communication network factor 1 (CCN1) as an important factor to induce fibroblast senescence and subsequent expression of senescence-associated anti-fibrotic genes [58]. Binding of CCN1 to integrin α6β1 induced an ROS-dependent DDR, activating the TP53 and the p16/RB pathways, thereby limiting fibrosis in cutaneous wounds. Furthermore, in 2015, a study by Shi et al. [59] reported a requirement for wingless type (Wnt) and NOTCH pathway activation for timely wound closure. The involvement of NOTCH signalling may implicate juxtacrine secondary senescence in the skin. In another study, transient exposure of keratinocytes to SASP led to the up-regulation of stemness markers and increased regenerative skin capacity *in vivo*, suggesting a beneficial effect of senescence mediators. However, prolonged exposure to SASP caused senescent cell cycle arrest and reduced regenerative capacity [60], again illustrating kinetic-dependent heterogeneity in the outcome.

In conclusion, wound healing and age-related skin senescence highlight the differences in senescence outcomes within the same tissue, with one senescence aspect — wound healing — being a desired outcome and another one — reduced epidermal regeneration — conferring a potentially pro-ageing effect. The timing of senescence might also lead to heterogeneity, where senescence-mediated stemness is promoted transiently, but regenerative capacity decreased by persistent senescence. In addition, studies in liver highlight the importance of the context and duration in which senescence occurs, with acute liver damage harvesting beneficial aspects of senescence which turn detrimental in the context of chronic damage. Overall, senescence might be initially beneficial in tissue context if it is properly controlled and senescent cells are cleared. However, where senescence persists over time, negative effects promoting disease and ageing take over.

## Functional senescence heterogeneity and response to senescence therapy

### Pro-senescence therapy

Senescence was originally described as a tumour suppressor pathway [2,3]. Therefore, in the context of anticancer therapies, senescence induction is one way to influence tumour progression. Senescence can be induced by drugs, such as Palbociclib, which engages with multiple senescence pathways. Palbociclib activates RB1 to induce cell cycle arrest and it up-regulates the proteasome, causing cellular stress due to increased proteolysis [61,62].

One of the main tumour suppressor pathways is the TP53 pathway. Therefore, targeting TP53 directly or indirectly to reactivate the pathway is conceptually another anticancer intervention. In this context, TP53 restoration in malignant liver cancer led to senescence induction and tumour clearance in the mouse [63]. Restoration of TP53 in pre-neoplastic, proliferating pineal lesions also induced cellular senescence in mice [63]. However, TP53 restoration in invasive pineal tumours did not induce senescence, illustrating that cancer stage, tissue type and timing of senescence induction are important and can lead to heterogeneous outcomes.

### Anti-senescence therapy

Senolytics are drugs that selectively eliminate senescent cells, often by targeting pro-survival pathways that increase resistance to apoptosis in senescent cells [64]. Several pro-survival networks have been identified relating to the PI3K/AKT and TP53 pathways and the BCL2 apoptosis regulator (BCL-2) family, varying with tissue and senescence inducing mechanism [64]. Many current senolytics act in a cell type or inducer specific manner. For example, Fisetin selectively eliminates senescent cells in umbilical vein endothelial cells (HUVECs), but not in human lung fibroblasts (IMR90s) or preadipocytes. On the other hand, A1331852 and A1155463 are senolytics effective in HUVECs and IMR90 cells, but not preadipocytes [65]. One of the most commonly studied senolytics targeting PI3K/AKT upstream of NF-κB, Dasatinib, eliminates human adipocyte progenitors, with a lesser effect on HUVECs [66]. In contrast, Querceptin, a potent antioxidant and metal ion chelator, has little effect on preadipocytes, but induces apoptosis in senescent HUVECs compared with growing cells. Finally, Navitoclax has been widely used in *in vivo* mouse models, effectively eliminating senescent cells in a variety of tissues. However, Navitoclax is ill tolerated and was initially suggested as a broad-spectrum senolytic, with lethal effects in HUVECs and IMR90 cells, but little effect on senescent preadipocytes [66].

Targeting the SASP through direct or indirect modulation of the NF-κB pathway has yielded some success in a wide variety of model systems [65,66]. For example, glucocorticoids, a group of steroid hormones including cortisol and corticosterone, have anti-inflammatory effects via down-regulation of NF-κB transcriptional activity [66]. Metformin is known to regulate glucose metabolism, but can also prevent NF-κB translocation to the nucleus, thereby reducing the SASP. Moreover, mTor inhibitors, such as rapamycin, interfere with the IL-1α/NF-κB axis, leading to reduced secretion of IL-6/8 [66]. Since IL-6/8 are important components of the SASP, these might act on a wide variety of senescent cells.

In summary, more specific senescence therapies should aim to eliminate the detrimental effects of specific senescence populations whilst not impacting on potential beneficial aspects of senescence. Therefore, careful understanding of the different outcomes of senescence with regards to the whole organism, and not just selective tissues, is necessary to enhance healthy life span, whilst promoting continued tissue repair and tumour suppression. A broad range of tissue-specific senescence markers need to be assessed during senescence therapy to gain a more complete picture.

## Perspectives

- Senescence is a highly heterogeneous and complex cellular phenotype, with different effects depending on tissue/cell of origin and senescence inducer. The interplay between primary and secondary senescence and how they differ in contributing to age-related disease and cancer outcomes are poorly understood. It is important to study the features of a heterogeneous senescent cell population with the aim to enhance beneficial aspects of senescence (tumour suppression, increase glucose production, wound healing) with simultaneous down-regulation of damaging senescence aspects (inflammatory cytokine release, decreased regeneration).
- Senolytics are drugs that selectively eliminate senescent cells, targeting pro-survival pathways that increase resistance to apoptosis in senescent cells. Pro-survival networks often vary with tissue and senescence inducing mechanism, with current senolytics often acting in a cell type or senescence inducer specific manner [64]. Senostatics, such as antioxidants, suppress paracrine senescence signalling without killing the senescent cells and might be useful in some contexts [67].
- Characterising heterogeneity in senescent cell populations creates the basis for effective senotherapy development. Therefore, the extent, interplay and consequences of primary and secondary senescence and functional senescence heterogeneity need to be urgently addressed.

## Open Access

Open access for this article was enabled by the participation of University of Edinburgh in an all-inclusive *Read & Publish* pilot with Portland Press and the Biochemical Society under a transformative agreement with JISC.

## Abbreviations

CCFs: cytoplasmic chromatin fragments
CCL2: chemokine CC motif ligand 2
CDKN1A: cyclin-dependent kinase inhibitor 1A
DAMP: damage-associated molecular patterns
DDR: DNA damage response
HDFs: human diploid fibroblasts
MMPs: metalloproteinases
RB1: Rb transcriptional co-repressor 1
ROS: reactive oxygen species
SAHF: senescence-associated heterochromatin foci
SASP: senescence-associated secretory phenotype
SA-β Gal: senescence-associated β galactosidase
TLR2: Toll-like receptor 2
TNF-α: tumour necrosis factor-alpha
TP53: Tumour protein 53
VEGF: vascular endothelial growth factor
